# Reduced Incidence of *Prevotella* and Other Fermenters in Intestinal Microflora of Autistic Children

**DOI:** 10.1371/journal.pone.0068322

**Published:** 2013-07-03

**Authors:** Dae-Wook Kang, Jin Gyoon Park, Zehra Esra Ilhan, Garrick Wallstrom, Joshua LaBaer, James B. Adams, Rosa Krajmalnik-Brown

**Affiliations:** 1 Swette Center for Environmental Biotechnology, Biodesign Institute, Arizona State University, Tempe, Arizona, United States of America; 2 Virginia G. Piper Center for Personalized Diagnostics, Biodesign Institute, Arizona State University, Tempe, Arizona, United States of America; 3 Department of Biomedical Informatics, Arizona State University, Scottsdale, Arizona, United States of America; 4 School for Engineering of Matter, Transport and Energy, Arizona State University, Tempe, Arizona, United States of America; 5 School of Sustainable Engineering and the Built Environment, Arizona State University, Tempe, Arizona, United States of America; Argonne National Laboratory, United States of America

## Abstract

High proportions of autistic children suffer from gastrointestinal (GI) disorders, implying a link between autism and abnormalities in gut microbial functions. Increasing evidence from recent high-throughput sequencing analyses indicates that disturbances in composition and diversity of gut microbiome are associated with various disease conditions. However, microbiome-level studies on autism are limited and mostly focused on pathogenic bacteria. Therefore, here we aimed to define systemic changes in gut microbiome associated with autism and autism-related GI problems. We recruited 20 neurotypical and 20 autistic children accompanied by a survey of both autistic severity and GI symptoms. By pyrosequencing the V2/V3 regions in bacterial 16S rDNA from fecal DNA samples, we compared gut microbiomes of GI symptom-free neurotypical children with those of autistic children mostly presenting GI symptoms. Unexpectedly, the presence of autistic symptoms, rather than the severity of GI symptoms, was associated with less diverse gut microbiomes. Further, rigorous statistical tests with multiple testing corrections showed significantly lower abundances of the genera *Prevotella*, *Coprococcus*, and unclassified *Veillonellaceae* in autistic samples. These are intriguingly versatile carbohydrate-degrading and/or fermenting bacteria, suggesting a potential influence of unusual diet patterns observed in autistic children. However, multivariate analyses showed that autism-related changes in both overall diversity and individual genus abundances were correlated with the presence of autistic symptoms but not with their diet patterns. Taken together, autism and accompanying GI symptoms were characterized by distinct and less diverse gut microbial compositions with lower levels of *Prevotella*, *Coprococcus*, and unclassified *Veillonellaceae*.

## Introduction

Autism Spectrum Disorders (ASD) are complex neurobiological disorders whose chief manifestations are qualitative impairment in social interaction and communication and restricted, repetitive, and stereotyped patterns of behavior, interests, and activities [1]. There is a world-wide increase in the diagnosis of ASD, which has reached an epidemic level [2]. ASD patients and their families face difficulties in treatment due to a highly diverse etiology of ASD in which genetic and environmental factors are equally important, as implied by a large twin concordance study [3]. One potentially important environmental factor is abnormal intestinal flora. A large fraction of autistic children suffer from gastrointestinal (GI) problems, and a strong positive correlation was observed between GI problems and ASD severity [4]. Up to 10^14^ bacteria in human intestine balance the immune system, help digestion, produce vitamins, and promote GI motility [5]. Several studies have reported an increased administration of oral antibiotics to autistic children during the first 3 years of life [6]–[8], which may destabilize microbial community by eliminating beneficial bacteria and helping pathogenic bacteria colonize the intestinal walls [9]. In addition, considering the potential interactions between intestinal microbes and the central nervous system [10], abnormal intestinal flora may be associated not only with GI problems but also with ASD-related behavioral symptoms.

Potential involvement of gut microbes in ASD etiology has been speculated for more than a decade. Many pathogenic gram-negative bacteria contain lipopolysaccharide (LPS) in their cell walls, which can cause damage in various tissues including the brain [11]. LPS-induced inflammation in the brain increases permeability of the blood-brain barrier and facilitates an accumulation of high levels of mercury in cerebrum, which may aggravate ASD symptoms [6]. A test in rats showed that prenatal LPS exposure decreased levels of glutathione [12], which is an important antioxidant involved in heavy metal detoxification in the brain. The down-regulated synthesis of glutathione may increase the vulnerability of children to ASD and other neurologic disorders, such as Friedreich’s Ataxia [13]. Indeed, a recent pyrosequencing analysis showed that gram-negative bacteria, *Desulfovibrio* and *Bacteroides vulgatus,* were detected at higher levels in autistic children [14]. On the other hand, *Clostridium*, a gram positive bacterium, has also been widely studied in the context of ASD [15], [16] because it produces exotoxins and propionate. In a study on rats which was recently reported, propionate worsened ASD-like behavior [17]. In addition, *Clostridium difficile* produces p-cresol, which can cause depletion of glutathione [18]. Vancomycin and ampicillin, antibiotics targeting cell wall damage, significantly affect the physiology and structure of gut microbiota; especially on gram-positive bacteria such as *Clostridium difficile* [19]. One study showed that in a small scale clinical setting, vancomycin treatment resulted in temporary improvement of autistic symptoms in children with late-onset autism [20].

Despite the importance of community-wide balance in maintaining healthy gut flora, there have been limited efforts to investigate gut microbiota as a functionally interconnected microecosystem in relation to autism. Moreover, previous studies describing the relationship between autism and gut microbes have either mostly focused on the emergence of harmful bacteria or mainly paid attention to already-known beneficial bacteria, such as *Bifidobacteria* and *Lactobacillus* [4], [14], [21]. In this study, we collected data on relative abundances of intestinal bacteria from neurotypical and autistic children by 454 Titanium pyrosequencing, and investigated systemic differences in their microbiome. Our comprehensive bioinformatics and statistical analyses demonstrated that autistic children have a distinct and less diverse gut microbial community structure, which can be characterized by lower levels of a group of distinctive bacterial genera.

## Materials and Methods

### Ethics Statement

The Institutional Review Board (IRB) at Arizona State University approved the study (ASU IRB Protocol #: 1004005109). People who expressed interest in joining our study were mailed a written consent form and initial questionnaires (Participation questionnaire and GI severity index form) to complete and return to us. Once we received written informed consent forms (signed by parents or guardians) and complete questionnaires, we mailed sample collection kits to prospective participants.

### Subject Recruitment and Sample Collection

We recruited 20 neurotypical children and 20 children with ASD. The enrolled subjects were between the ages of 3 and 16 years and did not use any type of antibiotic or antifungal medications at least within one month prior to sample collection. We assessed the GI symptoms of the children with a modified version of the GSI questionnaire [22]. Children with ASD were assessed with the Autism Diagnostics Interview – Revised (ADI-Revised), Autism Diagnostics Observation Schedule (ADOS), Autism Treatment Evaluation Checklist (ATEC), and Pervasive Developmental Disorder Behavior Inventory (PDD-BI) to ensure that they had a diagnosis of autism. We also conducted surveys documenting some of their diet patterns, such as: gluten-free/casein-free (GF/CF) diet, probiotics use, seafood consumption, and usage of nutrient supplements (*e.g*., vitamins/calcium). Detailed information on the survey can be found in the Supporting Information (Text S1).

#### Sample collection and DNA extraction

Parents collected and froze a single fecal sample from each subject. Frozen fecal samples were shipped overnight to Arizona State University with a cold pack, and stored in −80°C until DNA extraction. In order to target DNA that was heterogeneously distributed in stool samples, we followed the protocol recommended by QIAamp (Qiagen, CA) DNA Stool Mini Kit (Protocol: Isolation of DNA from larger volumes of stool). We first collected one gram (wet weight) of stool sample per extraction, added 10 ml ASL lysis buffer solution, and vortexed it vigorously until the stool sample was thoroughly homogenized. Then, we used 2 ml of the lysate and followed the manufacturer’s recommendations. We assessed the quantity and quality of DNA by measuring absorbance at 260 and 280 nm using a NanoDrop ND-1000 spectrophotometer (NanoDrop Technology, Rockland, DE). Before we sent genomic DNA samples to the sequencing facility for pyrosequencing, we confirmed PCR amplification with universal bacterial primers [23], and ran agarose gel (1%, w/v) electrophoresis to confirm the efficiency of PCR amplification visually.

### Pyrosequencing Analysis of Community Structures

We sent extracted genomic DNA to the Research and Testing Laboratory (Lubbock, TX, USA), where the bacterial tag-encoded FLX amplicon pyrosequencing (bTEFAP) was performed by the Genome Sequencer FLX-Titanium System and its Titanium protocol (Roche, Indianapolis, IN) as described [24]. We targeted V2 and V3 regions of 16S rDNA for pyrosequencing by selecting bacterial primers 104F (5′-GGCGVACGGGTGAGTAA-3′) and 530R (5′-CCGCNGCNGCTGGCAC-3′). We denoised PCR artifacts by eliminating sequences with low qualities [25]. Using SILVA Incremental Aligner (SINA) implemented in the Mothur software, we aligned qualified sequences to bacterial reference set identified from the SSURef database [26], and removed chimeric sequences by ChimeraSlayer [27]. We removed one outlier with a very low sequence count and microbial diversity. Then, we rarefied all the other samples by randomly sampling 15,951 sequences from each sample ten times in order to match the sequence numbers across samples. Either normalized sequence counts from individual subsamples or the average counts of ten subsamples were used for further analyses, as indicated in text for each analysis. To obtain the operational taxonomic units (OTUs), we clustered the sequencing readouts at 90, 95, and 97% similarity with UCLUST algorithm [28], which are roughly equivalent to the taxonomic terms of family, genus, and species, respectively. We used the Mothur software to obtain rarefaction curves and ecological indices of Chao1 estimator and Shannon diversity/richness [25]. We measured phylogenetic diversity (PD) index by using Quantitative Insights Into Microbial Ecology (QIIME) software package [29]. We classified sequences by the RDP Classifier software at the 50% and 80%-confidence threshold for sequence length longer than 200 basepair (bp) and 250 bp, respectively [30]. We also performed the phylogeny-based metric UniFrac analysis in order to evaluate the distribution of *Prevotella* species [31]. Detailed analysis pipeline is provided in the Supporting information (Text S1).

### Quantitative Real-time PCR Analysis

We performed 16S rDNA-targeting quantitative real-time PCR (qPCR) with triplicate PCR reactions in an Eppendorf Realplex 4S RealCycler. For *Prevotella* species, we constructed a seven-point standard curve by using genomic DNA of *Prevotella copri* (DSM18205). The PCR reagent mixture for each reaction was 20 µL consisting of 8 µL of 2.5× SYBR Premix Ex Taq Mix (Takara Bio Inc, Japan), 1 µL of 10 µM *Prevotella*-specific each forward and reverse primers [32], 2 ul 10-fold diluted DNA as a template, and 8 µL PCR grade water. The PCR amplification was conducted with initial 10 minute denaturation at 95°C, followed by 35 cycles of denaturation (95°C for 15s), and annealing/extension (60°C for 60s). For general bacteria, we performed qPCR following the protocols described in Ziv-El et al. [33].

### Statistical and Data Analysis

Statistical tests and classifications were done by Python (SciPy stats library), and R programming (p.adjust for multiple testing correction; glm for logistic regression; the glmperm package for permutation test for regression [34]; the randomForest package for Random Forests classification [35]). Hierarchical clustering (average linkage) was performed with the Biopython package, and clustergrams were generated by the Reportlab package for Python (ver.2.6.5). Receiver operating characteristics (ROC) curves and the area under the ROC curve (AUC) values were obtained using the caTools package in R. PCA was performed using the prcomp function (scaled and centered) in R, from which the coordinates for genus and samples were obtained. Canonical correlation analysis (CCA) and permutation test were conducted by using the CCP package in R.

## Results

### Subject Characteristics

We collected stool samples from neurotypical and autistic subjects (n = 20 each after gender balancing), with the mean ages (± SD) of 8.3 (±4.4) and 6.7 (±2.7) years, respectively (Table 1 and Dataset S1). To estimate the severity of GI symptoms, we surveyed 6 categories of GI Severity index (6-GSI) (Table 1). As previously reported, all autistic subjects had some degrees of GI disorders [4]. However, when the severity of GI problems was compared with autism severity, the 6-GSI scores did not have strong correlations with any index for autistic symptoms, including the ADOS (Pearson/Spearman rank correlation coefficient r = −0.33/0.28), the ATEC (r = 0.25/0.26), and the PDD-BI (r = 0.15/0.09). We also found no statistical significance (Fisher transformation and permutation tests P>0.05, Table 2).

**Table 1:** Summary of subject characteristics.

Subject characteristic	Neurotypical	Autistic
Total # participants	20	20
Male/Female	17/3	18/2
Age (years)	8.3±4.4	6.7±2.7
ADOS	–	12.7±3.9
ATEC	–	71.5±23.4
PDD-BI	–	−52.8±50.0
6-GSI	0.5±0.8	4.7±2.3
# of subjects using GF/CF diet	1	5
# of subjects using nutritional supplements	8	13
Probiotics (per week)	1.6±2.6	3.3±3.4
Seafood (per week)	0.4±0.5	1.2±2.3

Detailed information including GI severity, autistic severity indices, and diet survey is attached in the Dataset S1.

**Table 2:** Correlations between the severity of GI problems and autism severity indices.

	Age	ADOS	PDDBI	ATEC
Pearson (r value)	−0.147	0.33	0.145	0.252
P_1_/P_2_	0.55/0.74	0.17/0.08	0.58/0.29	0.31/0.16
Spearman rank (r value)	−0.141	0.277	0.089	0.257
P_1_/P_2_	0.57/0.73	0.25/0.12	0.74/0.37	0.30/0.16

Pearson and Spearman rank correlation between GI severity scores with autism severity (P1: Fisher transformation, P2: permutation test).

### Autism-associated Changes in Intestinal Microbial Diversity

**Figure 1:**
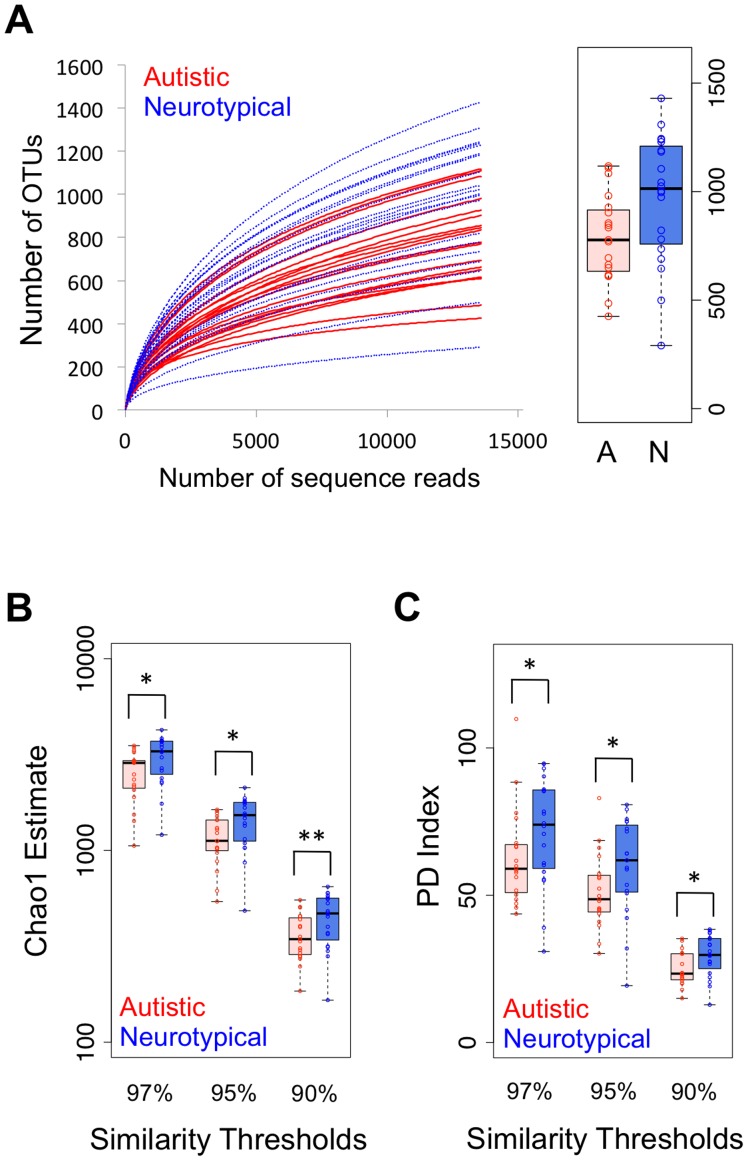
Comparison on bacterial richness and diversity between neurotypical and autistic children. (A) Rarefaction curves showing unique OTUs at the 95% threshold (a box graph at the rarefied sequence number), comparison of (B) Chao1 estimators and (C) phylogenetic diversity (PD) index between neurotypical (blue-colored box) and autistic (red-colored box) groups at different similarity thresholds (*: P<0.05, **: P<0.01 by one-tailed Mann-Whitney test).

Maintaining sufficient bacterial richness and diversity is important for providing gut microbiota with functional redundancy, adaptability, and thus systematic robustness against environmental challenges [36]. With a total of 987,801 qualified sequences, we rarified each sample at 15,991 reads in order to estimate bacterial richness and diversity (Table S1). Rarefaction curves showed that neurotypical individuals had a higher number of observed bacterial species than autistic individuals (Figure 1A and Figure S1). As an alternative method to estimate the richness, we employed the nonparametric Chao1 estimator in various levels of OTU clustering. Similar to the rarefaction data from observed OTUs, the neurotypical group had significantly higher number of estimated OTUs (Figure 1B and Table S2A), which indicates that the neurotypical group had higher bacterial richness than the autistic group. To estimate microbial diversity, we employed two ecological indices: Shannon and Phylogenetic Diversity (PD). First, Shannon measurements showed a similar trend to the Chao1 estimator, but without statistic significance (Table S2B). The Shannon index combines both bacterial richness and evenness. The similar microbial evenness between samples (Table S2C) offset the significance of bacterial richness when we calculated the Shannon index. Different from the Shannon index, the PD index incorporates the concept of phylogenetic distinctiveness and is sensitive to species relatedness by summing the tree-branch length present in each subject of a phylogenetic dendrogram [37]. The PD index revealed that the neurotypical group harbored more diverse gut microbiota than the autistic group did (P<0.05 by one-tailed Mann-Whitney test in Figure 1C and Table S2D). In addition, we also evaluated correlation between bacterial richness/diversity and the severity of GI problems within the autistic group, and bacterial richness was negatively correlated with GI severity (Table S3). Taken together, these analyses suggest that the presence of autistic symptoms and their correlated GI problems are linked with reduced richness and diversity of gut microflora, which results in a decrease in microbial redundancy and alter the physiological functionality and microbial GI robustness in children with ASD.

To investigate if autism-linked microbiome changes were associated with other variables, such as general demographics and special diets, we first performed multivariate analyses. Our results indicate that age, gender, and diet were not significant factors determining ecological indices (P>0.05) (Table S4), but Chao1 estimator was most significantly correlated with autism status (P<0.05) (Table 3 and Table S4). We also conducted canonical correlation analysis (CCA) followed by permutation-based non-parametric significance tests. As shown in Table S4C, the combined profiles of diversity indices were not confounded by demographics and/or diet patterns (P>0.05). Multivariate analyses and CCA results show that reduced richness in children with autism was not due to demographics or special diets.

**Table 3:** Permutation-based tests on univariate and multivariate regression models with the autistic status and microbial diversity indices.

		P value		Univariate			Multivariate		
Diversity index	UCLUSTthreshold	Student’st-test	Mann-Whitney	Coef	P	SE	Coef	P	SE
Chao	97%	0.014	0.008	0.00	0.017	0.004	0.00	0.012	0.003
	95%	0.010	0.008	0.00	0.018	0.004	0.00	0.011	0.003
	90%	0.013	0.010	−0.01	0.028	0.005	−0.01	0.008	0.003
Shannon	97%	0.120	0.080	−0.62	0.202	0.013	−1.29	0.075	0.008
	95%	0.295	0.120	−0.25	0.598	0.016	−0.60	0.310	0.015
	90%	0.420	0.100	−0.09	0.847	0.011	−0.29	0.583	0.016
Shannon-even	97%	0.250	0.140	−3.53	0.494	0.016	−10.2	0.147	0.011
	95%	0.460	0.210	0.40	0.922	0.008	−2.89	0.568	0.016
	90%	0.350	0.380	1.12	0.702	0.014	−0.41	0.905	0.009
PD	97%	0.062	0.031	−0.03	0.097	0.009	−0.04	0.151	0.011
	95%	0.039	0.021	−0.04	0.066	0.008	−0.05	0.102	0.010
	90%	0.018	0.021	−0.11	0.031	0.005	−0.13	0.066	0.008

(Coef: coefficient from linear regression, P: P value from permutation likelyhood ratio test for generalized linear model, SE: standard error for P value).

### Autism-associated Changes in Gut Microflora at Phylum Level

For detailed taxonomic analyses, individual sequences were classified by the RDP classifier [30], which assigned approximately 97% of total sequences to 15 known phyla. *Firmicutes and Bacteroidetes* were the two most dominant phyla, as previously reported by many studies [38], and the phyla *Proteobacteria, Verrucomicrobia*, and *Actinobacteria* were also relatively abundant (Figure S2). These five phyla comprised an average of 97.2% of total classifiable sequences across samples. Comparison of mean abundances between groups by the Student’s t-test showed that the phyla *Proteobacteria* and *Verrucomicrobia* were more abundant in neurotypical than autistic groups, respectively, but without statistical significances after correction for multiple testing (Table S5). Since the abundance data were not normally distributed and contained a large fraction of zero values, we employed the non-parametric Mann-Whitney test, which was used as the main statistical test for comparisons throughout this study. The tests showed that there was no significant difference in the relative abundance of individual phyla between the two groups.

### Autism-associated Changes in Gut Microflora at the Genus Level

**Figure 2:**
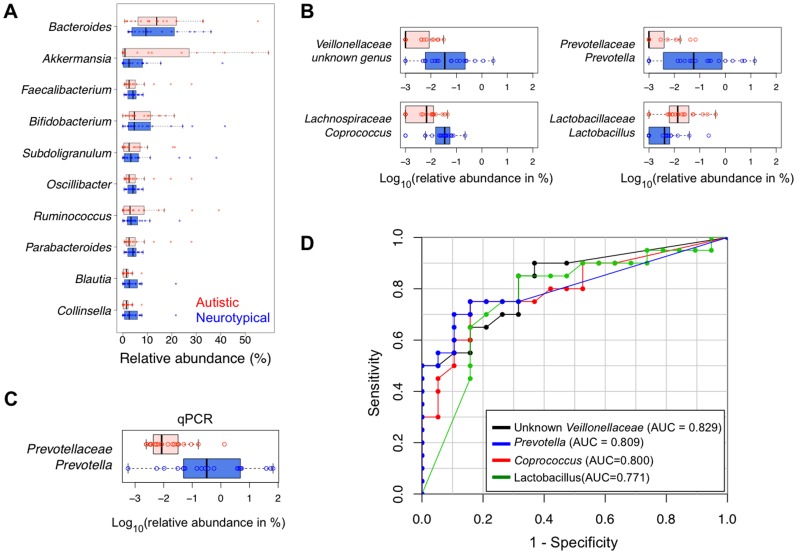
Distribution of 39 subjects based on relative abundance. (A) The top 10 most abundant genera, (B) 4 most differentially abundant genera (Red-colored box: autistic children, blue-colored box: neurotypical children), (C) the genus *Prevotella* obtained by qPCR analysis (Red-colored box: autistic children, blue-colored box: neurotypical children), and (D) receiver operating characteristics (ROC) curve of the 4 genera that have the highest area under curve (AUC).

Among 214 genera identified by the RDP classifier, the genera *Bacteroides, Faecalibacterium, Bifidobacterium, Akkermansia, and Subdoligranulum* were the top 5 most abundant genera in both neurotypical and autistic groups. The 5 genera comprised 40% and 56% of total sequences, respectively (Figure 2A and Table S6). Interestingly, the genus *Akkermansia* was present at very high levels in several autistic subjects, representing up to 59% of all sequences, and the prevalence in those samples contributed to low diversity (Figure S3).

The mean abundance of each genus was compared between groups by the Student’s t-test and Mann-Whitney test (Table S7 and Table S8). The Mann-Whitney test with multiple testing correction showed that unclassified *Veillonellaceae* and *Prevotella* were significantly more abundant in the neurotypical group than in the autistic (adjusted P = 0.04, Table S8, Figure 2B). In addition, with marginal statistical significances, abundance of *Coprococcus* and unclassified *Prevotellaceae* were also higher in neurotypical samples (adjusted P = 0.06, Table S8, Figure 2B). To verify our interesting results for *Prevotella*, we performed qPCR and confirmed the reduced abundance in autistic children (Mann-Whitney test P = 0.0002, Figure 2C). In order to measure how correctly the relative abundance of each genus could classify two groups of samples, we employed the ROC curve, which is closely related to the Mann-Whitney test and commonly used to evaluate classification performance of potential biomarkers. The performance of a given binary classifier can be evaluated by measuring the AUC that depicts true versus false positive rates, where an AUC value of 0.5 corresponds to random classification and a value of 1.0 corresponds to perfect classification. The above-mentioned genera (unclassified *Veillonellaceae*, *Prevotella*, and *Coprococcus* together with *Lactobacillus*) showed the highest AUC values among all genera, up to 0.829 (Figure 2D and Table 4). We also obtained high AUC values from parallel classifiers. Using six of the top ten genera in terms of AUC, we constructed a logistic regression classifier and measured its predictive capability using leave-one-out cross-validation. The cross-validation AUC was 0.839, and inclusion of age and gender into the logistic regression classifier did not improve predictive performance. We also constructed a classifier using Random Forests technique, and observed a similar performance (cross-validation AUC = 0.837). The AUC values from above classifiers were highly comparable to those of known biomarkers of many clinical disorders, *e.g*., urine biomarkers for drug-induced kidney injury [39] and blood-based markers for myocardial infarction [40].

**Table 4:** Top 10 genera generating the highest area under curves in a receiver operating characteristics curve.

		Mann-Whitney		Median (25%/75%)	
Family. Genus	AUC	P	P adj.	Neurotypical	Autistic
*Veillonellaceae.unknown_genus*	0.829	0.000	0.039	0.04(0.01/0.21)	0(0/0.01)
*Prevotellaceae.Prevotella*	0.809	0.001	0.039	0.06(0.01/0.67)	0(0/<0.01)
*Lachnospiraceae.Coprococcus*	0.800	0.001	0.062	0.03(0.02/0.05)	0.01(0/0.01)
*Lactobacillaceae.Lactobacillus*	0.771	0.003	0.096	<0.01(0/<0.01)	0.01(<0.01/0.04)
*Prevotellaceae.unknown_genus*	0.754	0.002	0.062	<0.01(0/0.02)	0(0/0)
*Alcaligenaceae.Sutterella*	0.746	0.004	0.096	0.06(0/0.18)	0(0/0)
*Lachnospiraceae.Roseburia*	0.746	0.009	0.139	0.06(0.03/0.18)	0.02(0.01/0.05)
*Porphyromonadaceae.Butyricimonas*	0.712	0.007	0.139	0.01(0/0.09)	0(0/0)
*Eubacteriaceae.Eubacterium*	0.709	0.026	0.277	0.03(0.02/0.10)	0.13(0.05/0.25)
*Prevotellaceae.Paraprevotella*	0.703	0.009	0.139	<0.01(0/0.35)	0(0/0)

A highest area under curves (AUC) value of 0.5 indicates no predictive, while an AUC of 1 indicates perfect ability to predict.

In addition, we also performed permutation-based tests on multivariate regression models to assess whether GI problems or other clinical factors such as age, gender, dietary intakes, and autism severity indices had any effect on the relative abundance of the top ten genera generating the highest AUC values. Tests on all samples showed that age, gender, and dietary intakes were not significantly associated with the genus abundances (Table S4A), but the autistic status turned out to be the most significantly correlated factor with the abundances of these genera, especially with unclassified *Veillonellaceae*, *Prevotella*, and *Coprococcus* (P<0.05 in Table 5 and Table S4A). A mantel test (CCA) also supported that age, gender, ethnicity, and dietary intake, when tested separately or in combination, were not significantly correlated with the abundance profile of the ten most differentially present genera between the subject groups (P>0.1 in Table S4C). Furthermore, within the autistic group, we did not observe any significant correlation between age, gender, severity of GI symptoms, as well as autistic indices, and the differentially present genus-level abundances (Table S4B). Together, these multivariate analyses demonstrate that there is a statistically significant correlation between the presence of autistic symptoms and the genus-level abundances and that severity of GI symptoms is not a significant predictor of these microbial changes among autistic children.

**Table 5:** Permutation-based tests on univariate and multivariate regression models with the autistic status and top 10 genera generating the highest AUC.

	Univariate			Multivariate		
Family. Genus	Coef	P	SE	Coef	P	SE
*Veillonellaceae.unknown_genus*	−0.36	0.000	0.000	−0.53	0.002	0.001
*Prevotellaceae.Prevotella*	−0.19	0.000	0.000	−0.17	0.001	0.001
*Lachnospiraceae.Coprococcus*	−0.44	0.000	0.000	−0.56	0.001	0.001
*Lactobacillaceae.Lactobacillus*	0.05	0.171	0.012	0.02	0.742	0.014
*Prevotellaceae.unknown_genus*	−0.03	0.060	0.008	−0.06	0.085	0.009
*Alcaligenaceae.Sutterella*	−0.04	0.021	0.005	−0.08	0.034	0.006
*Lachnospiraceae.Roseburia*	0.00	0.244	0.014	0.00	0.494	0.016
*Porphyromonadaceae.Butyricimonas*	−0.07	0.087	0.009	−0.05	0.297	0.014
*Eubacteriaceae.Eubacterium*	0.02	0.071	0.008	0.01	0.787	0.013
*Prevotellaceae.Paraprevotella*	−0.03	0.017	0.004	−0.06	0.004	0.002

(Coef: coefficient from linear regression, P: P value from permutation likelyhood ratio test for generalized linear model, SE: standard error for P value).

### Species-level Analyses of Prevotella

**Figure 3:**
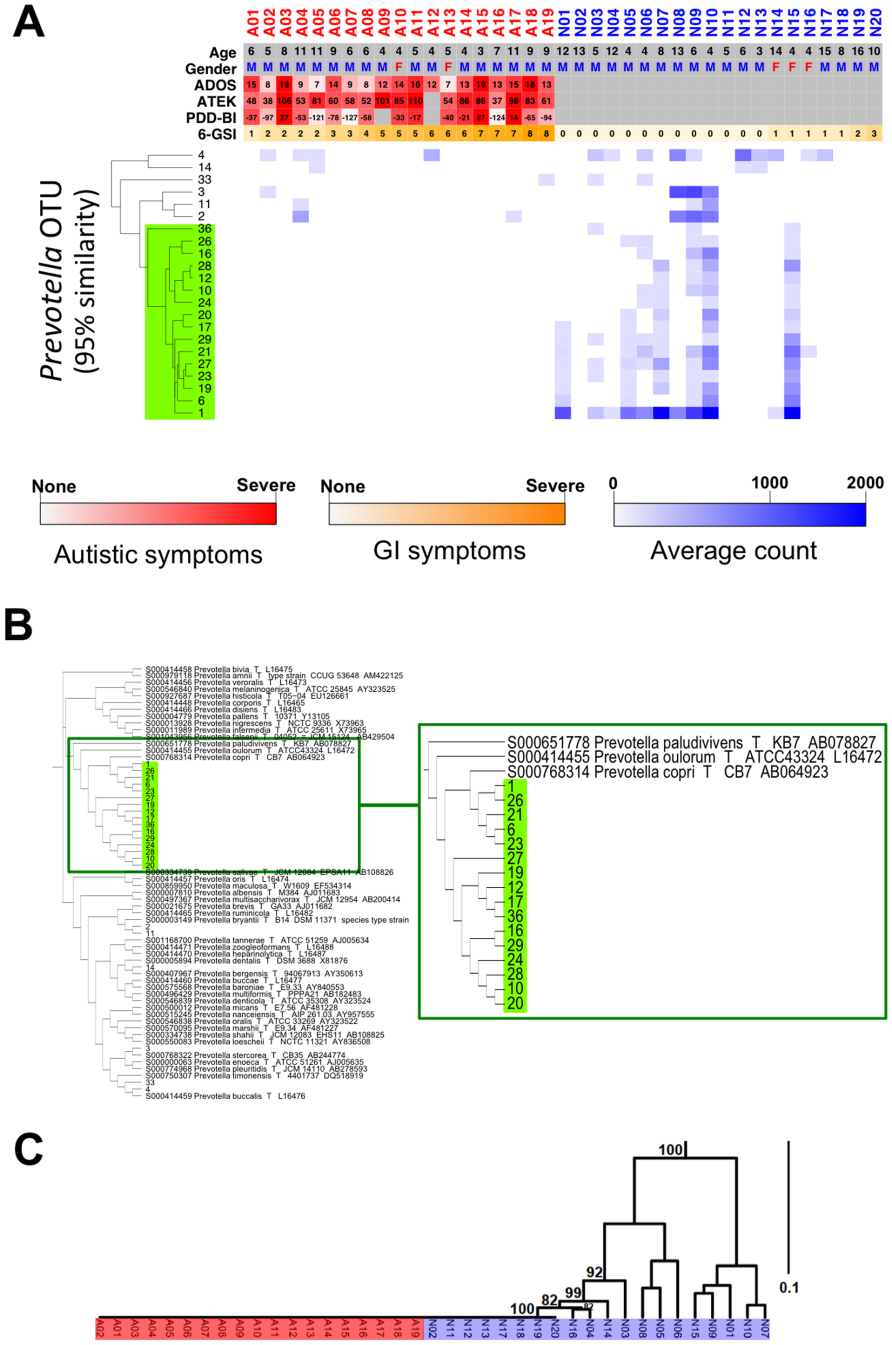
Comparison of gut microbiota within the genus *Prevotella* between neurotypical and autistic children. (A) Heat map profile and dendrogram (A01-A19: autistic children, N01–N20: neurotypical children). A red, orange, and blue scale bar represents scores of autistic symptoms, GI problems, and a log scale of the percentile abundance from a total bacteria, respectively. (B) Phylogenetic tree within the genus *Prevotella*. (C) The weighted UniFrac analysis with *Prevotella* copri-like 16 OTUs. Jackknife counts over 50 out of 100 are shown.

A majority of 16S rDNA-based metagenomic analyses have been done at a genus level. However, the observed abundance profile of any given genus may represent the sum of heterogeneous species and/or strains, which can lead to a less accurate interpretation. Therefore, we investigated whether we could obtain in-depth inference from the sub-genus level analyses. Among fully classified genera, *Prevotella* showed the most significant differences between the sample groups by various statistical tests (Figure 2D, Table 4, and Table S8). In addition, indicating the physiological importance, a series of recent metagenomic studies suggests that *Prevotella* is a key genus in determining gut microbiome profile [41], [42]. Thus, we decided to further investigate *Prevotella* at a sub-genus level. First, all sequence reads that were classified by RDP as the genus *Prevotella* were re-classified into discrete OTUs at a 95% similarity level by UCLUST. The 22 identified OTUs were then clustered based on their relative abundance across samples (Figure 3A). Noticeably, while the other OTUs showed a scattered distribution across both groups, a major cluster of 16 OTUs was exclusively present in neurotypical samples (Figure 3A, in the green box). In order to see if the cluster of 16 OTUs represented any known species, we combined the 16 OTU sequences with 16S rDNA sequences of 42 known type-cultured *Prevotella* species and performed a phylogenic analysis by multiple alignments. The dendrogram in Figure 3B shows that 16 *Prevotella* OTUs had higher sequence similarities to *Prevotella copri* that was reported as the most prevalent *Prevotella* species in the gut of North American population [43]. When we cross-matched the OTUs between two independently generated clusters (*i.e*. based on their relative abundance vs. sequence similarities), all 16 OTUs in the major cluster identified in Figure 3A were mapped to the sequence cluster that contained *P. copri* (Figure 3B, in the green zoomed box). With these *Prevotella copri*-like 16 OTUs, we also performed a weighted UniFrac analysis and observed distinct clustering patterns between neurotypical and autistic groups (Figure 3C). In addition, analyses on the co-occurrence and co-exclusion network demonstrated that the loss of *Prevotella copri*-like OTUs in autistic samples could result in a major reconfiguration as well as a reduced complexity in the intestinal microbial ecosystem (Figure S4). Taken together, these data strongly showed that autistic children have very low levels of the *Prevotella copri*-like phylotype in their GI tracts and that this phylotype is more frequently found in neurotypical children who have more diverse and robust microbial consortia.

### Autism-associated Changes in Microbial Community Profile and Gut Enterotypes

**Figure 4:**
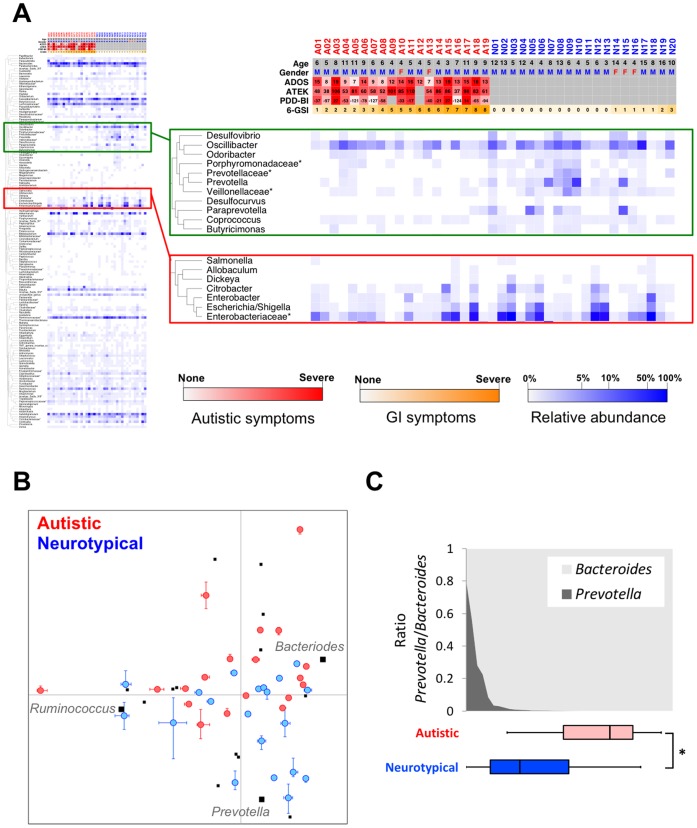
Genus level comparison of gut microbiota between neurotypical and autistic children. (A) Heat map profile and dendrogram of all identified genera (A01-A19: autistic children, N01–N20: neurotypical children). A red, orange, and blue scale bar represents scores of autistic symptoms, GI problems, and a log scale of the percentile abundance from a total bacteria, respectively. (B) Principal Component Analysis at the genus level from the autistic and neurotypical children. Blue- and red-, and black-colored dots represent neurotypical, autistic samples, and 16 selected genera, respectively. Three genera representing enterotypes (23) were identified in bold. (C) The gradient of *Prevotella* and *Bacteroides* through neurotypical and autistic children (*: P<0.05 by Mann-Whitney test).

Like other environmental microbial ecosystems, human intestinal microflora is distinctively shaped by diverse microorganisms and their mutual interactions. Therefore, autism-related microflora alterations could be found at the level of community rather than at the individual microbe level. In an effort to identify the systematic differences in microbial communities between neurotypical and autistic groups, all genera were hierarchically clustered based on their relative abundance across samples (Figure S5). While the majority of clusters showed no apparent difference between the groups, one cluster of 11 genera that included *Prevotella* was, in general, at a greater abundance in neurotypical samples (Figure 4A, green box). In addition, these genera shared a similar pattern, especially within neurotypical samples, indicating a coherent relationship among them. Interestingly, we also noticed that another cluster enriched in *Enterobacteriaceae* (Figure 4A, red box) displayed a negatively correlated pattern to the *Prevotella* cluster within the neurotypical group (Pearson/Spearman rank correlation coefficient r = −0.52/−0.67, Fisher transformation test P = 0.02/0.001, and permutation test P = 0.0002/0.0008). However, we did not observe an increase of the *Enterobacteriaceae* cluster in autistic children despite the significant decrease in *Prevotella*; suggesting that the community-wide interrelationship of gut microbiota was altered in autistic children.

Recently, global profiles of the human gut microbiome were examined to identify central components of the microbial community [41], [42]. Notably, *Prevotella* was proposed as one of the three main microbes representing the human gut microbiome, namely enterotypes [41]. Further, the ratio gradient between *Prevotella* and *Bacteroides* was reported to be a good ecological metric for characterizing gut microbiota in adults [42]. Therefore, given that the presence as well as abundance of *Prevotella* was the main difference between neurotypical and autistic groups, we tested whether any systemic change in the gut microbiome profile was associated with autism in relation to the enterotype and *Prevotella/Bacteroides* gradient. First, we performed a principal component analysis (PCA) on all 39 samples with the relative abundance of 16 selected genera (Table S9) that were reported in the co-occurrence network of Arumugam *et al.* [41]. Although the PCA analysis did not show three clearly discrete groups with the core genera (*i.e*. *Prevotella*, *Bacteriodes,* and *Ruminococcus*), we observed that *Prevotella*, *Bacteriodes*, and *Ruminococcus* were among the main contributors of the first and the second principal components (Table S10). When the frequencies of these pre-defined enterotypes were compared between groups, the *Prevotella*-rich enterotype was absent in the autistic group, while neurotypical samples showed an even distribution among the three enterotypes (Figure 4B). We also examined whether autistic subjects had different trade-off patterns between *Prevotella* and *Bacteroides* at the genus level, and observed that distributions of autistic and neurotypical groups were clearly distinguishable by the *Prevotella/Bacteroides* gradient (Figure 4C). In summary, these data demonstrate that the autistic population is associated with a gut microbial community profile that is distinct from that of a representative human population.

## Discussion

Given the crucial role of gut microorganisms in maintaining GI health, increasing evidence of more frequent occurrence of GI problems in autistic children strongly implies a link between autism and gut microbiota. Although the direction of causality among interconnected pathophysiological factors (*e.g.,* autistic symptoms, diet patterns, GI symptoms, and gut microbiome profile) is still unclear, it is important to identify systemic microbiome changes and specific microorganism(s) that can be targeted for diagnosis as well as for treatment of autism-related GI problems and possibly other autistic symptoms. As a first step to reach this long-term goal, we discovered several key differences. First, autistic children tend to have a less diverse gut microbiome. Secondly, several individual genera, most notably *Prevotella*, are found at significantly lower abundances in autistic children. Lastly, there are autism-associated global changes in intestinal microbial community.

In our dataset, autistic samples displayed relatively low microbial richness and diversity, especially when the relatedness or phylogenetic distinctness was conjointly considered with abundance for diversity estimation, such as the PD index (Table S2) [37], [44]. Higher diversity of gut bacteria may allow better microbial integrity and the ability to protect the human intestine from environmental stresses such as intake of pathogenic gut microbes [45]. De Filippo et al. [46] observed higher diversity of gut bacteria in rural African children than European children,and hypothesized that the fiber-rich diet of African children provided greater resistance to GI disorders, such as diarrhea than low-fiber diet of European children. A metagenomic study by Qin et al. [47] found 25 percent fewer genes in the gut of patients with irritable bowel syndrome than in healthy controls. In contrast, Finegold et al. [14] found a higher microbial diversity in autistic children and suggested that an increase of pathogenic bacteria might worsen autistic symptoms.

Discrepancies among high-throughput 16S rDNA sequencing studies are common and can be resulted from several reasons, such as sample source (*e.g*., feces vs. mucosal biopsy), uneven coverage of microbes by different PCR primers, and the number of sequence reads. In theory, based on our *in silico* simulation of 16S rDNA-based PCR on entire microbiome, commonly used primer sets for each variable region covered only 40 to 80% of known genera (*unpublished data*). Moreover, the source of samples may be an important contributor for the discrepancies. For example, Finegold *et al* [14] reported a decreased *Firmicutes* to *Bacteroidetes* ratio in autistic fecal samples, whereas Williams *et al* [48] observed an opposite trend in ileum and cecum biopsy samples from neurotypical children than those from autistic children, both with GI problems. However, we observed no significant difference of *Bacteroidetes* and *Firmicutes* between neurotypical and autistic children. Additionally, analysis of biopsy samples showed that the genus *Sutterella* in the *Alcaligenaceae* family was predominant in autistic children with GI problems compared to neurotypical children with GI problems [21]. We, however, observed that *Sutterella* was slightly less abundant in fecal samples from autistic children compared to the neurotypical ones (adjusted P = 0.096, Table 4). In addition to the differences in the experimental setting, the type and stringency of statistical tests, such as consideration of false discovery rates for multiple testing conditions, might affect the interpretation of data.

Among fully classified genera in our study, *Prevotella* showed the most significant difference in relative abundance between autistic and neurotypical subjects. *Prevotella* is increasingly gaining attention as a commensal microbe in the human large intestine because of its ability to degrade a broad spectrum of plant polysaccharides [49]. *Prevotella* species were highly prevalent in African children whose diet is rich in grains [46], implying not only that *Prevotella* plays a key role in digesting carbohydrate-rich food, but also that diet patterns can affect the abundance of *Prevotella* in human gut. Supporting these notions, Wu et al. [49] recently reported that carbohydrate-based diets shifted intestinal microbiota towards the *Prevotella*-rich enterotype. Interestingly, autistic children are known to have significant deficiencies in dissacharide metabolism, for example, a low level of lactase activity in upper GI tract [48], [50]. Williams et al [48] noted that unabsorbed mono- and disaccharides may enter into the large intestine and cause the imbalance of gut enviroment, where mono- and disaccharides fermenters may outcompete polysaccharide degrading *Bacteroidetes* species, such as *Prevotella*. *Prevotella* species also have essential genes for biosynthesis of vitamin B1 [41], which was reported to mitigate ASD symptoms [51]. Similar to our observation, Finegold et al [14] also recorded the depletion of *Prevotella* in autistic children in contrast to its prevalence in sibling controls, but no further discussion with statistical analysis followed this observation. A significantly lower abundance of the *Prevotella* cluster in autistic children could be linked to a low carbohydrate diet, however, autistic and neurotypical children generally consume comparable amounts of carbohydrate and fiber [52]. In addition, Huws et al [53] reported *Prevotella* as one of the dominant gut microbes enriched by fish oil supplementation in the diet of ruminants. Fish oil is a precursor of omega-3 fatty acids, which are beneficial for normal brain development [54]. However, our data showed no significant correlation between *Prevotella* abundance and the frequency of seafood consumption. To clarify the relationship between diet patterns and gut microbiota in autistic children, further studies with detailed diet diaries are warranted.

In addition to its physiological functions as an individual microbe, accumulating evidence supports a central niche of *Prevotella* in maintaining the community structure of human gut microbiome, as highlighted in studies by Yatsunenko et al [42] and Arumugam et al. [41]. Generally agreeing with the enterotype study [41], we observed similar interrelationships between *Prevotella* and its neighboring bacteria in our co-occurrence network. For example, the *Prevotella*-cluster in Figure 4A contains unclassified *Veillonellaceae*, *Coprococcus*, *Desulfovibrio,* and *Oscillibacter*. Unclassified *Veillonellaceae* genus was not dominant in either neurotypical or autistic groups, but turned out to be one of the most significantly different genera between the two groups (Figure 2D). Most members of the family *Veillonellaceae* (for example, *Veillonella* and *Megasphaera*) ferment lactate [55], [56], which may explain a potential metabolic link between *Veillonella* and *Prevotella* as both genera *Veillonella* and *Prevotella* also appeared in the co-occurrence network [41]. Other studies show that the abundance of the family *Veillonellaceae* increased when polydextrose and soluble corn fiber were part of an adult diet [57]. However, in equine large intestines, abundance of *Veillonellaceae* remained constant even with diets with increased levels of sugar and starch [58]. *Coprococcus* species are also fermenting bacteria, which mainly produce butyrate [59], [60]. Further implying a potential collaborative physiological function of the *Prevotella* cluster as a group, Arumugam et al. [41] speculated that *Desulfovibrio* species work synergistically with *Prevotella* species to degrade mucin. In addition, *Desulfovibrio*, *Prevotella*, *and Oscillibacter* can utilize microbial exopolysaccharides synthesized by *Bifidobacterium* to produce short-chain fatty acids in the human intestine [61]. Finegold et al [14] claimed that *Desulfovibrio* was significantly more abundant in autistic children compared with control children. However, they used a less stringent statistical testing approach in which no multiple testing correction was performed to control false discovery. In addition, the inconsistent observation on *Desulfovibrio* can be partly explained by different primer sets for pyrosequencing. We observed that the relative abundance of *Desulfovibrio* was not significantly different between neurotypical and autistic groups after multiple testing correction (Table S8), but its co-occurrence with other genera in the *Prevotella* cluster may suggest a potentially beneficial physiological role in metabolism, in contrast to the pathogenic role speculated by Finegold et al [62].

As previously reported [41], [46], we observed an *Enterobacteriaceae*-rich cluster containing potentially pathogenic bacteria such as *Salmonella, Escherichia/Shigella,* and *Citrobacter* [63]. The *Enterobacteriaceae* cluster displayed an opposite trend to the *Prevotella* cluster among the neurotypical subjects (Figure 4A). De Filippo et al. [46] suggested that suppression of pathogenic *Escherichia/Shigella* by *Prevotella* might result in a lower incidence of GI disorders among African children. Interestingly, unlike within the neurotypical group, we did not observe this negatively correlated relationship between the two clusters within the autistic group in which both groups of bacteria were found at very low levels. This suggests a substantial change in the balance of the gut microbiome in autistic children.

Since all autistic subjects in this study had certain degrees of GI problems, it is possible that the observed microbial differences are associated with the presence of GI symptoms. However, the GI indexes were not correlated with the changes in microbiome profiles among autistic children (Table S4). Moreover, the balance between three enterotypes appeared to be maintained even in the presence of inflammatory bowel disease and obesity [41] that are known to profoundly alter gut microbiome [64], [65]. Moreover, a recent study reported that irritable bowel syndrome (IBS) subjects had a different pattern of enterotypes, where the *Ruminococcus*-rich enterotype was highly dominant [66], whereas autistic samples contained evenly distributed *Ruminococcus*-rich and *Bacteroides*-rich enterotypes (Figure 4B). Interestingly, Rajilic-Stojanovic et al. [66] also reported that IBS samples had moderately reduced levels of several *Prevotella* species (*P. oralis*, *P. ruminicola*, and *P. tannerae*). In agreement, these species were observed at slightly lower levels in autistic samples than neurotypical ones in our study (Figure 3B). However, an abundance of *P. copri*-related *Prevotella* species, such as *P. oulorum* and *P. paludivivens*, was reduced in autistic subjects (Figure 3) but not in IBS patients [66]. Therefore, distinct from other GI disorders, autism-related GI disorders may be linked to a unique shift in microbial balance, reflected by the absence of *Prevotella*, especially the *P. copri* -like phylotype.

Despite its experimental versatility and well-established bioinformatics resources for phylogenetic classification, 16S rDNA-based metagenomic studies have an intrinsic limitation for biological inference. Due to functional redundancy and crosstalk among microbes [43], it is difficult to predict inclusive physiological impact of microbiome changes simply by measuring presence/absence and abundance of bacteria. Metabolic outcomes can be bioinformatically estimated by overlaying the total gene content in genomes of identified microbes onto known pathway information, as previously attempted in metagenomic studies [41], [42], [49]. However, in order to obtain an in-depth view on functional crosstalk between host and microbial composition changes, more comprehensive data including metatranscriptome, proteome, and metabolome are needed.

The long-term effect of enhanced antibiotics use is a potentially important factor that has not been addressed in this and other previous autism-related metagenomic studies. Studies focusing on fecal metabolites and microbiota show that gut microbiota and its diversity can be restored and stabilized within a month after antibiotic treatments [67]–[69]. Therefore, in this study, we only included subjects who had not taken any antibiotics at least within a month prior to sample collection. Thus, we assumed that the gut microbiota in the collected fecal samples had already recovered and stabilized after recent or past antibiotic treatment. Notably, in a recent paper by Dethlefsen and Relman [68], *Prevotella*, one of the significant genera observed here, was re-established back to pre-antibiotic levels within one month after ciprofloxacin treatment. However, we cannot rule out the possibility that more frequent exposure to antibiotics at earlier ages may perturb gut microbiota for a longer term in autistic children [70] even though autistic and neurotypical children take antibiotics at similar frequencies after the age of three [8]. Further retrospective and longitudinal studies are essential to accurately assess the long-term influence of antibiotics use in relation to autism.

In summary, we demonstrated that autism is closely associated with a distinct gut microflora that can be characterized by reduced richness and diversity as well as by altered composition and structure of microbial community. Most notably, we also discovered that the genera *Prevotella*, *Coprococcus*, and unclassified *Veillonellaceae* were significantly reduced in autistic children. Unexpectedly, these microbial changes were more closely linked to the presence of autistic symptoms rather than to the severity of GI symptoms and specific diet/supplement regimens. Despite limited information on the direction of causality among autism, diet, GI problems, and microbiome profiles, the findings from this study are stepping stones for better understanding of the crosstalk between gut microbiota and autism, which may provide potential targets for diagnosis or treatment of neurological as well as GI symptoms in autistic children.

## Supporting Information

Figure S1 **Rarefaction curves to show sequencing numbers and observed operating taxonomic units (OTUs) obtained by UCLUST.** Sequence similarity thresholds at (a) 97%, (b) 95%, and (c) 90%.
(PDF)

Figure S2 **Relative abundance of gut microbiome at the phylum level**. Red boxes: autistic children, blue boxes: neurotypical children.
(PDF)

Figure S3 **Distribution of relative abundance of the genus** ***Akkermansia*** **in 39 subjects.** N: neurotypical, A: autistic group.
(PDF)

Figure S4 **Co-occurrence network at the 97% OTU level**. OTU pairs that show positive correlation (Pearson’s R >0.8) among normal samples are presented as a network. The edge colors represent correlation of each OTU pair among autistic samples, as indicated in legend. *Prevotella copri* like OTUs and their direct edges are highlighted in yellow and green, respectively, to show the extent of network perturbation resulted from the absence of the OTUs in autistic subjects.
(PDF)

Figure S5 **Heat map profiles and dendrograms of all identified genera**. A01–A19: autistic children, N01–N20: neurotypical children. The color represent averaged relative abundances in a log scale from 10 random sub-samplings.
(PDF)

Table S1 **Pyrosequencing data summary with the number of a total sequence reads and qualified sequence reads.** Subject description: Neurotypical (N) and Autistic (A) children.
(XLSX)

Table S2 **Microbial diversity indices with OTUs obtained by UCLUST (90, 95, and 97% similarity).** (A) Chao1 estimator, (B) Shannon diversity index, (C) Shannon evenness index, and (D) Phylogenetic Diversity. (P-values were obtained by one-tailed Student’s t-test and Mann-Whitney test.)
(XLSX)

Table S3 **Correlation between microbial richness/diversity and severity of GI problems within the autistic group.** (A) Chao1 estimator, (B) Shannon diversity index, (C) Shannon evenness index, and (D) Phylogenetic diversity. (P1 and P2: P values from Fisher transformation and permutation test).
(XLSX)

Table S4 **Permutation-based tests on univariate and multivariate regression models between the autistic status/demographic/diet patterns and microbial diversity indices/top 10 genera with the highest AUC (A) among all samples and (B) within autistic samples.** The coefficient for age, autism severity indices (ADOS, ATEK, PDD-BI), and GI index are the log odds ratios of genus abundance for a one unit change in the age, autism severity indices, and GI index, respectively. The coefficient for gender is the log odds ratio of genus abundance for males compared to females. P-values less than 0.05 are indicated in bold characters. (Coef: coefficient from linear regression, P: P value from permutation likelyhood ratio test for generalized linear model, SE: standard error for P value). (C) P values by permutation tests of canonical correlation analysis (CCA).
(XLSX)

Table S5 **Relative abundance (Median values (%) and 25/75 percentiles) of 15 phyla detected throughout all subjects.**
(XLSX)

Table S6 **Top 20 abundant genera among 214 known genera in neurotypical (N) and autistic (A) subjects.** The subject numbers (#N and #A) are counts of subjects that contain corresponding genera in fecal samples. The average percentage of each genus is indicated in the column ‘% Total.’
(XLSX)

Table S7 **Genera presenting significant difference between neurotypical and autistic children by two-tailed Student’s t-test results**. Relative abundance is shown as median values and 25/75 percentiles. Adjusted P values were 0.606 for all listed genera.
(XLSX)

Table S8 **Genera presenting significant difference between neurotypical and autistic children by nonparametric ranksum test** (two-tailed Mann-Whitney test). Relative abundance is shown as median values and 25/75 percentiles.
(XLSX)

Table S9 **The list of bacterial groups that presented in the co-occurrence networks of the enterotype study** (Arumugam et al. 2011)*. The genera in bold were used for our PCA analysis. The rest of genera were not considered because of no/little observation through our samples.
(XLSX)

Table S10 **List of genera considered for principal component analysis.** Main contributors of the first and second principal components are indicated in bold characters.
(XLSX)

Text S1 **Supplementary Materials and Methods.** Detailed information includes subject recruitment and characteristics and pyrosequencing analysis of community structure.
(PDF)

Dataset S1 **Detailed information of metadata on the survey of neurotypical and autistic participants.** Metadata include GI severity, autistic severity indices, diet survey, and relative abundances of gut microbiota at the genus level.
(XLSX)
