# Supplementary material for: Reduced Incidence of Prevotella and Other Fermenters in Intestinal Microflora of Autistic Children
Source: PLoS One. 2013 Jul 3;8(7):e68322. doi: 10.1371/journal.pone.0068322 (PMC3700858; doi:10.1371/journal.pone.0068322)
Supplement: Figure S1 — Rarefaction curves to show sequencing numbers and observed operating taxonomic units (OTUs) obtained by UCLUST. Sequence similarity thresholds at (a) 97%, (b) 95%, and (c) 90%. (PDF) [file pone.0068322.s001.pdf]

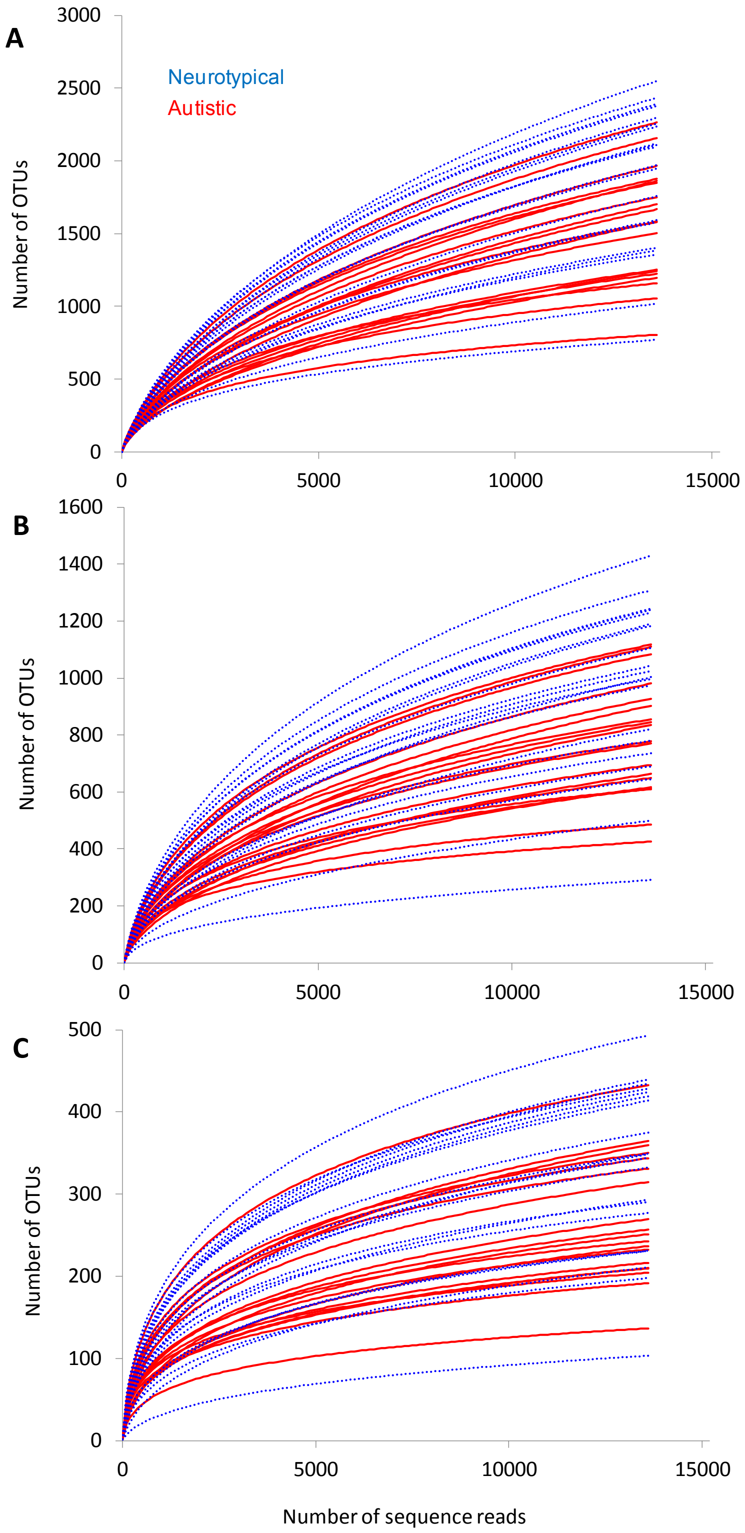

Fig. S1. Rarefaction curves to show sequencing numbers and observed operating taxonomic units (OTUs) obtained by UCLUST. Sequence similarity thresholds at (a) 97%, (b) 95%, and (c) 90%.
