# Supplementary material for: Reduced Incidence of Prevotella and Other Fermenters in Intestinal Microflora of Autistic Children
Source: PLoS One. 2013 Jul 3;8(7):e68322. doi: 10.1371/journal.pone.0068322 (PMC3700858; doi:10.1371/journal.pone.0068322)
Supplement: Figure S2 — Relative abundance of gut microbiome at the phylum level. Red boxes: autistic children, blue boxes: neurotypical children. (PDF) [file pone.0068322.s002.pdf]

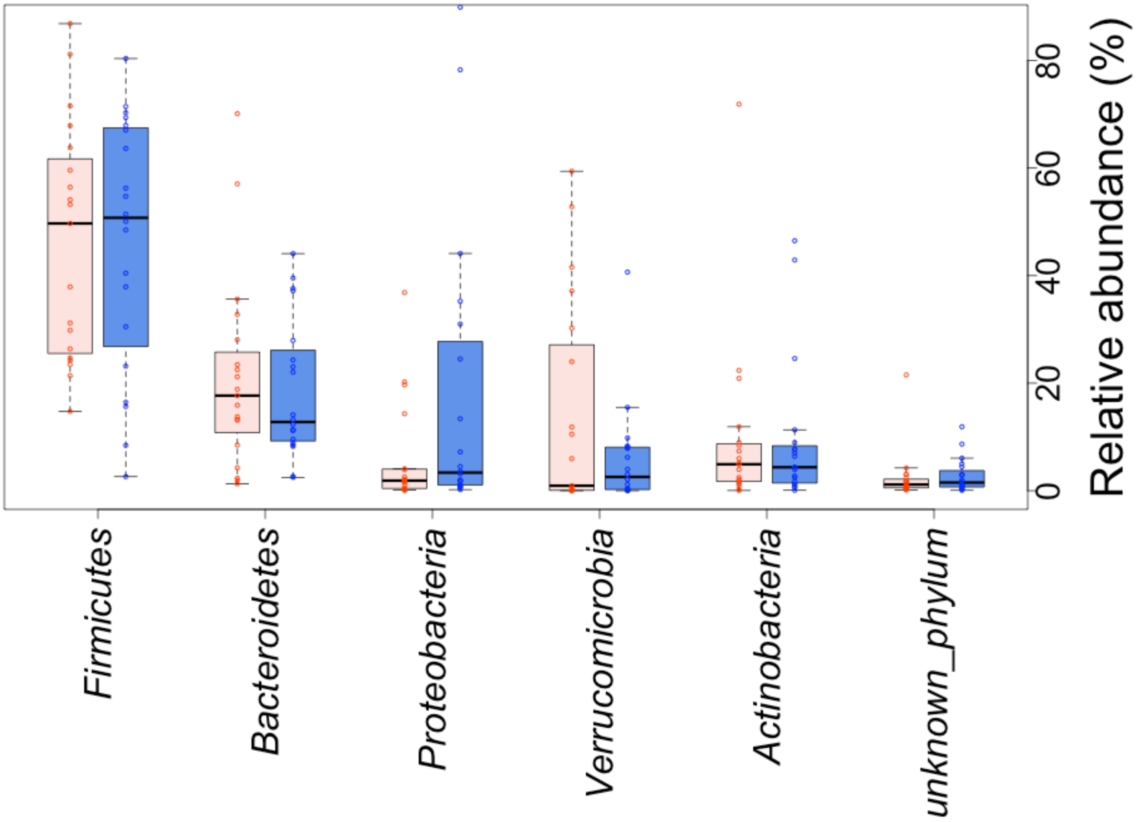

Fig. S2. Relative abundance of gut microbiome at the phylum level. Red boxes: autistic children, blue boxes: neurotypical children.
