# Supplementary material for: Reduced Incidence of Prevotella and Other Fermenters in Intestinal Microflora of Autistic Children
Source: PLoS One. 2013 Jul 3;8(7):e68322. doi: 10.1371/journal.pone.0068322 (PMC3700858; doi:10.1371/journal.pone.0068322)
Supplement: Figure S3 — Distribution of relative abundance of the genus Akkermansia in 39 subjects. N: neurotypical, A: autistic group. (PDF) [file pone.0068322.s003.pdf]

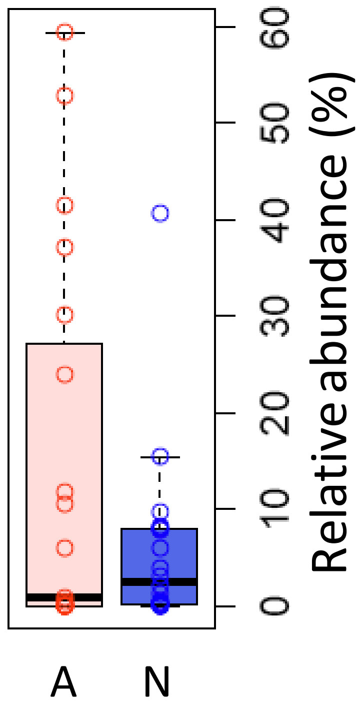

Fig. S3. Distribution of relative abundance of the genus *Akkermansia* in 39 subjects. N: neurotypical, A: autistic group.
