# Supplementary material for: Reduced Incidence of Prevotella and Other Fermenters in Intestinal Microflora of Autistic Children
Source: PLoS One. 2013 Jul 3;8(7):e68322. doi: 10.1371/journal.pone.0068322 (PMC3700858; doi:10.1371/journal.pone.0068322)
Supplement: Figure S4 — Co-occurrence network at the 97% OTU level. OTU pairs that show positive correlation (Pearson’s R >0.8) among normal samples are presented as a network. The edge colors represent correlation of each OTU pair among autistic samples, as indicated in legend. Prevotella copri like OTUs and their direct edges are highlighted in yellow and green, respectively, to show the extent of network perturbation resulted from the absence of the OTUs in autistic subjects. (PDF) [file pone.0068322.s004.pdf]

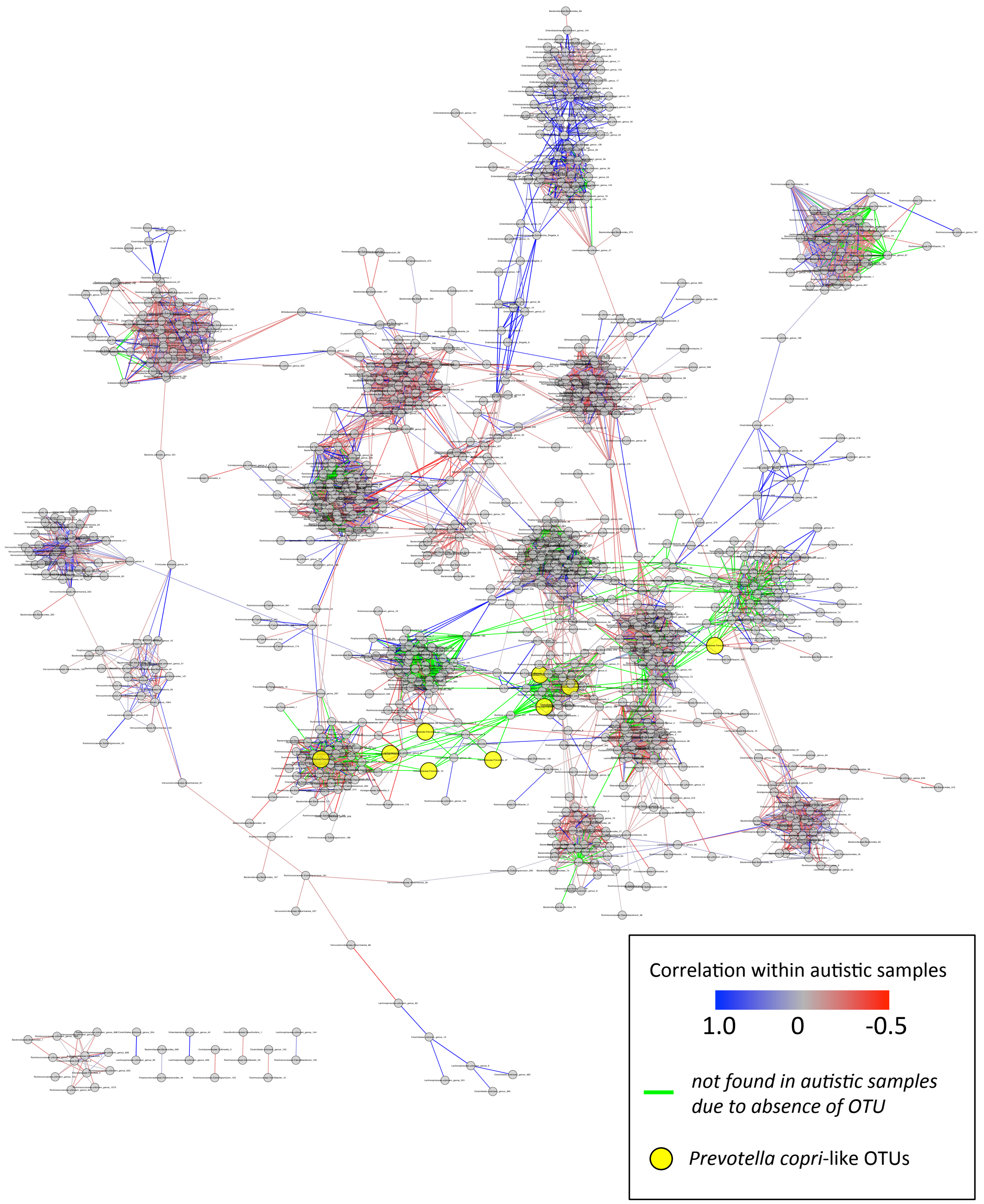

Fig. S4. Co-occurrence network at the 97% OTU level. OTU pairs that show positive correlation (Pearson's R > 0.8) among normal samples are presented as a network. The edge colors represent correlation of each OTU pair among autistic samples, as indicated in legend. Prevotella copri like OTUs and their direct edges are highlighted in yellow and green, respectively, to show the extent of network perturbation resulted from the absence of the OTUs in autistic subjects.
