# Supplementary material for: Reduced Incidence of Prevotella and Other Fermenters in Intestinal Microflora of Autistic Children
Source: PLoS One. 2013 Jul 3;8(7):e68322. doi: 10.1371/journal.pone.0068322 (PMC3700858; doi:10.1371/journal.pone.0068322)
Supplement: Figure S5 — Heat map profiles and dendrograms of all identified genera. A01–A19: autistic children, N01–N20: neurotypical children. The color represent averaged relative abundances in a log scale from 10 random sub-samplings. (PDF) [file pone.0068322.s005.pdf]

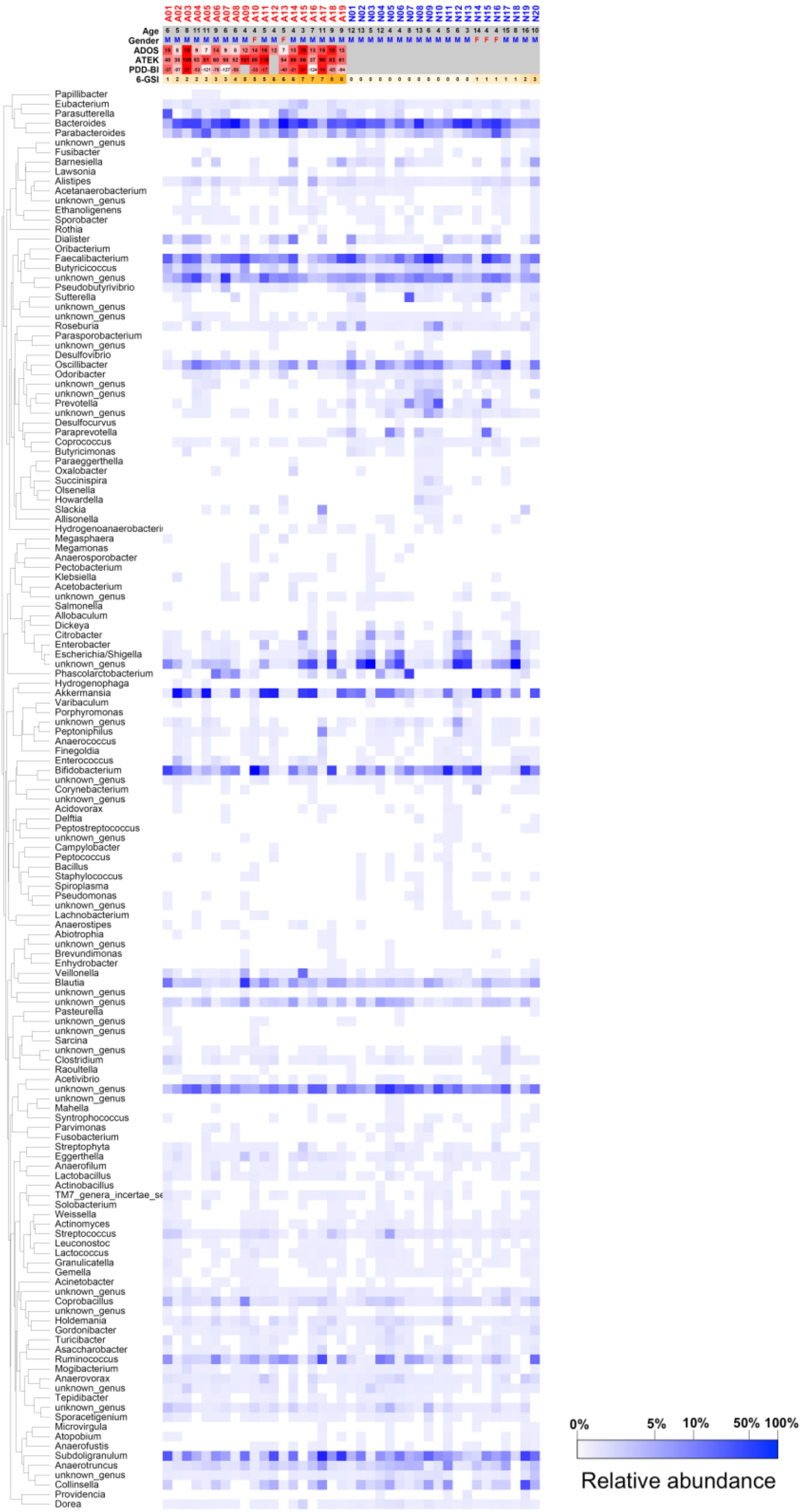

Fig. S5. Heat map profiles and dendrograms of all identified genera. A01-A19: autistic children, N01-N20: neurotypical children. The color represent averaged relative abundances in a log scale from 10 random sub-samplings.
