# Supplementary material for: Reduced Incidence of Prevotella and Other Fermenters in Intestinal Microflora of Autistic Children
Source: PLoS One. 2013 Jul 3;8(7):e68322. doi: 10.1371/journal.pone.0068322 (PMC3700858; doi:10.1371/journal.pone.0068322)
Supplement: Text S1 — Supplementary Materials and Methods. Detailed information includes subject recruitment and characteristics and pyrosequencing analysis of community structure. (PDF) [file pone.0068322.s016.pdf]

Supporting Information

SI Materials and Methods

Subject recruitment and characteristics. We enrolled fifty-six (56) applicants, which included 30 neurotypical and 26 autistic subjects. Eligibility criteria for subjects consist of age between 2 ½ to 17 years and no usage of any type of antibiotic or antifungal medications within one month. Neurotypical children that were first-degree relatives of children with ASD were excluded. The ATEC consists of four subscales: 1) speech/language/communication, 2) sociability, 3) sensory/cognitive awareness, and 4) health/physical behavior. The total ATEC score is the sum of the scores from each subscale. For PDD-BI scores, we determined a modified "Autism Composite" based on the addition of scores from three subscales-'sensory/perceptual approach behaviors', 'ritualisms/resistance to change', and 'social pragmatic problems', and subtracted scores from 'social approach behaviors' and 'expressive language'. Higher ATEC and PDD-BI scores indicate more severe ASD. ADOS consists of communication, social, play/imagination creativity, and stereo behavior/restrict interests scales, and we used the summation of communication and social scores to determine ASD.

We also assessed the gastrointestinal symptoms of the children with a modified version of the Gastro-Intestinal Severity index (GSI) questionnaire (Schneider et al 2006). Among GSI subscales, in our survey, we included six categories of symptoms (constipation, diarrhea, stool consistency, stool smell, flatulence, and abdominal pain). Each category had a 3-point scale, and summed up the points to get the total 6-GI Severity Index (6-GSI). The excluded subscales were 'unexplained daytime irritability', 'nighttime awakening', and 'abdominal tenderness.'

Out of 30 neurotypical subjects enrolled, one subject was excluded because of improper sample shipment, and 9 female children were not included to balance the number of gender with autistic children. The ratio of boys to girls with autism is found to be very high up to 6.5-to-1 (Johnson and Myers 2007), and similarly, we also ended up recruiting a small number of girls with autism. 3 female children selected for the study had little GI problems (6-GSI score was less than 2). Out of 26 autistic subjects enrolled, we excluded six children in our data evaluation: 1) two children who did not meet the ADOS described criteria, 2) two children who received antibiotic/antifungal treatment during the previous month, 3) one child who did not suffice to

submit required information, and 4) one child who dropped out. We did not constrain GI scores when we excluded six autistic children. The final 40 participants are listed in Table S1.

Pyrosequencing analysis of community structure. We obtained sequences by Genome Sequencer FLX-Titanium System (Roche, Indianapolis, IN) as described (Sun et al 2011). We selected bacterial primers 104F (5′-GGCGVACGGGTGAGTAA-3′) and 530R (5′-CCGCNGCNGCTGGCAC-3′) to amplify the combined V2 and V3 regions of 16S rDNA, and the amplicon was sequenced by the procedure described by Wolcott et al. (2009). We eliminated sequences having ambiguous basepair (bp), more than 2 primer and 1 barcode mismatches, homopolymers of more than 8 bps, an average quality score lower than 25, and shorter than 200 bp. Using SILVA Incremental Aligner (SINA) implemented in the Mothur software, we aligned qualified sequences to bacterial reference set identified from the SSURef database (Pruesse et al 2007), and then removed chimeric sequences by ChimeraSlayer (Haas et al 2011). Out of 40 samples with a total 987,801 non-chimeric sequences, one autistic sample with significantly fewer sequences (8,830 reads) than the average (24,695 reads) and with much lower diversity was excluded (Supplementary Table S2). We randomly subsampled the lowest number (15,991 sequence reads per sample) ten times, combined all sequences, and clustered sequences at 90, 95, and 97 % similarity by using UCLUST algorithm (Edgar 2010). We removed sequences that appeared only once across all samples. 10 different combined OTU tables were generated, and the average counts from OTU tables were used for further analysis.

For UniFrac analysis at the species level of *Prevotella*, we aligned representing sequences using SILVA-based alignments implemented in the Mothur software (Pruesse et al 2007), and constructed phylogenetic trees using FastTree2 (Price et al 2009). With Dendroscope (Huson et al 2007), we generated the tree files in the nexus format compatible to the online Fast UniFrac pipeline (http://bmf2.colorado.edu/fastunifrac/). Uploaded trees were analyzed with weighted Unifracs distances between samples. The Fast UniFrac online application calculated the UniFrac metric that measures the difference of unique branch lengths between samples, and provided phylogenetic analyses of ‘Cluster samples’. We ran the Jackknife Sample Clusters using 100 permutations for weighted analysis.

References

Edgar RC (2010). Search and clustering orders of magnitude faster than BLAST. *Bioinformatics* **26:** 2460-2461.

Haas BJ, Gevers D, Earl AM, Feldgarden M, Ward DV, Giannoukos G *et al* (2011). Chimeric 16S rRNA sequence formation and detection in Sanger and 454-pyrosequenced PCR amplicons. *Genome Res* **21:** 494-504.

Huson DH, Richter DC, Rausch C, Dezulian T, Franz M, Rupp R (2007). Dendroscope: An interactive viewer for large phylogenetic trees. *BMC Bioinformatics* **8**.

Johnson CP, Myers SM (2007). Identification and evaluation of children with autism spectrum disorders. *Pediatrics* **120:** 1183-1215.

Price MN, Dehal PS, Arkin AP (2009). FastTree: computing large minimum evolution trees with profiles instead of a distance matrix. *Mol Biol Evol* **26:** 1641-1650.

Pruesse E, Quast C, Knittel K, Fuchs BM, Ludwig WG, Peplies J *et al* (2007). SILVA: a comprehensive online resource for quality checked and aligned ribosomal RNA sequence data compatible with ARB. *Nucleic Acids Res* **35:** 7188-7196.

Schneider CK, Melmed RD, Enriquez FJ, Barstow LE, Ranger-Moore J, Ostrem JA (2006). Oral human immunoglobulin for children with autism and gastrointestinal dysfunction: A prospective, open-label study. *J Autism Dev Disord* **36:** 1053-1064.

Sun Y, Wolcott RD, Dowd SE (2011). Tag-encoded FLX amplicon pyrosequencing for the elucidation of microbial and functional gene diversity in any environment. In: Kwon YM, Ricke SC (eds). *High-throughput next generation sequencing: methods and application*. Humana Press. pp 129-141.

Wolcott RD, Gontcharova V, Sun Y, Dowd SE (2009). Evaluation of the bacterial diversity among and within individual venous leg ulcers using bacterial tag-encoded FLX and Titanium amplicon pyrosequencing and metagenomic approaches. *BMC Microbiol* **9**.
